# Carbamazepine‐induced Stevens‐Johnson syndrome in a patient with history of methotrexate‐induced mast cell activation syndrome

**DOI:** 10.1002/ccr3.3509

**Published:** 2020-11-11

**Authors:** Tamer Mohamed Zaalouk, Zouheir Ibrahim Bitar, Ossama Sajeh Maadarani, Mohamed Elsayed Elhabibi

**Affiliations:** ^1^ Critical Care Unit Ahmadi Hospital Kuwait Oil Company Ahmadi Kuwait

**Keywords:** carbamazepine, mast cell activation syndrome, methotrexate, Stevens‐Johnson syndrome

## Abstract

Stevens‐Johnson syndrome (SJS) is serious conditions that happen as a result of infection, side effects to medications, or unknown etiology. Carbamazepine is one of the common medications that can cause SJS. Good history taking is crucial if treatment with carbamazepine is clinically indicated. We would like to alert all physicians that carbamazepine should be avoided in any patient with a previous history of drug reaction such as mast cell activation syndrome.

## Graphical abstract

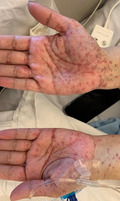

## 1 CASE REPORT

**FIGURE 1:**
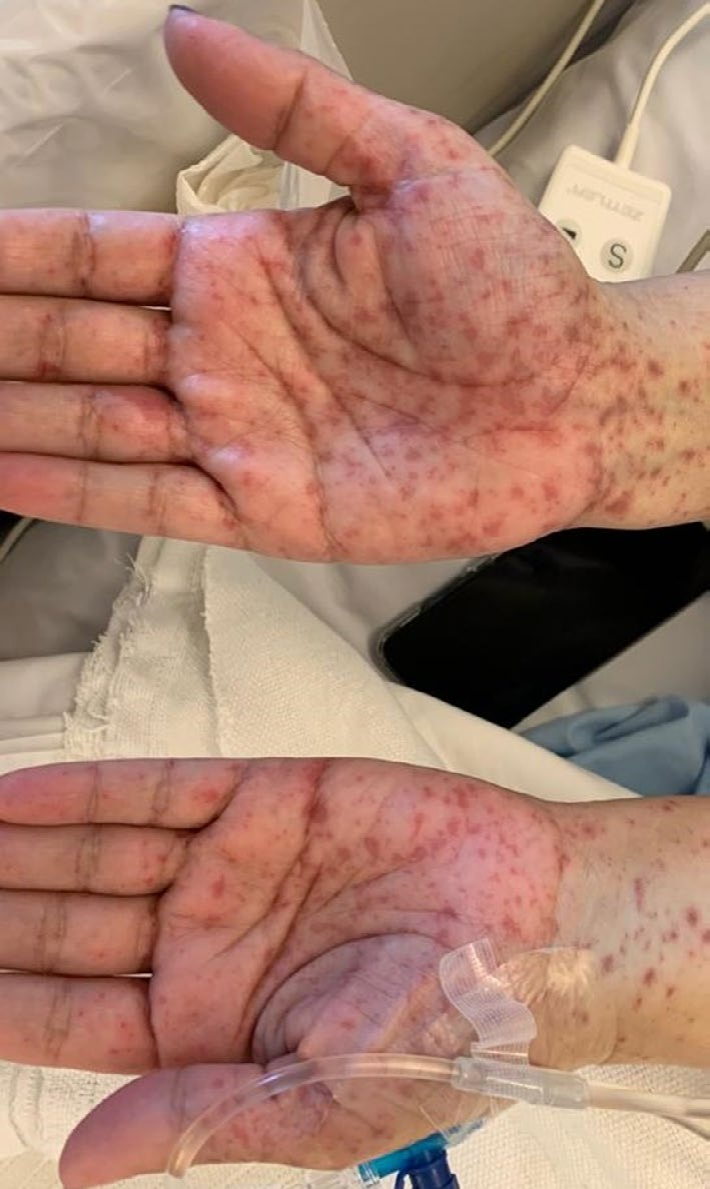
Generalised maculopapular rash on both hands

**FIGURE 2:**
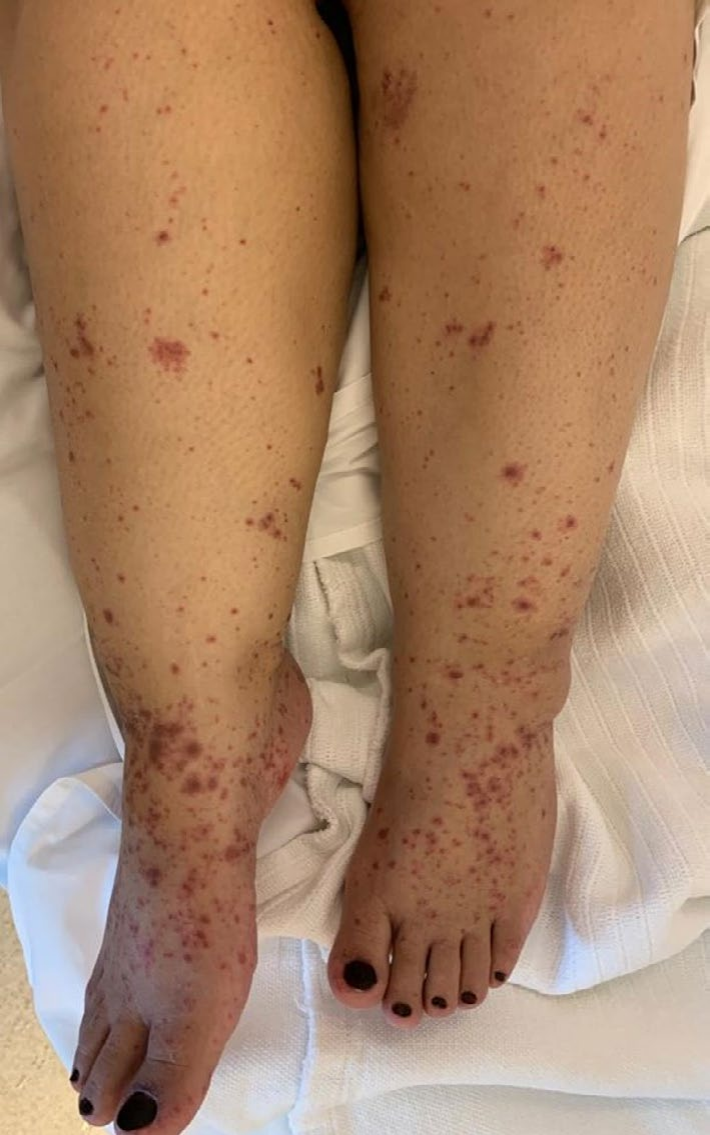
Generalised maculopapular rash with target lesions on lower limbs

**FIGURE 3:**
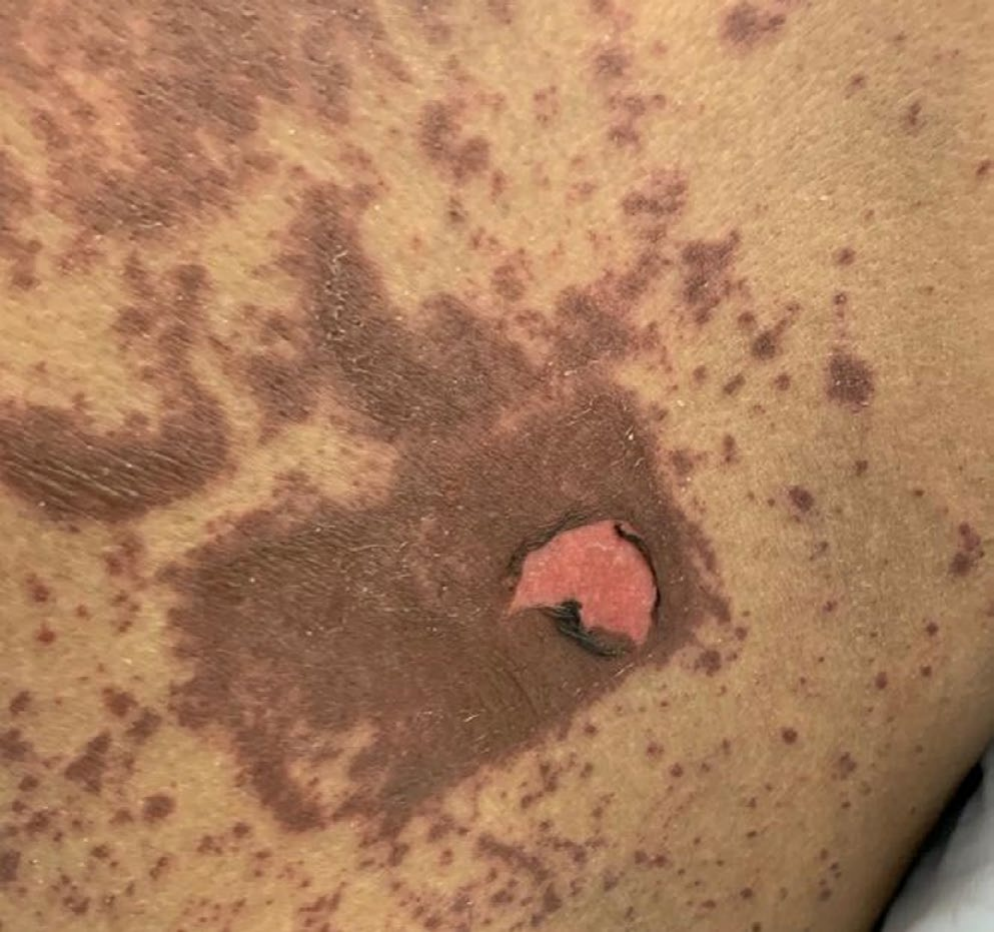
Nikolsky's sign

**FIGURE 4:**
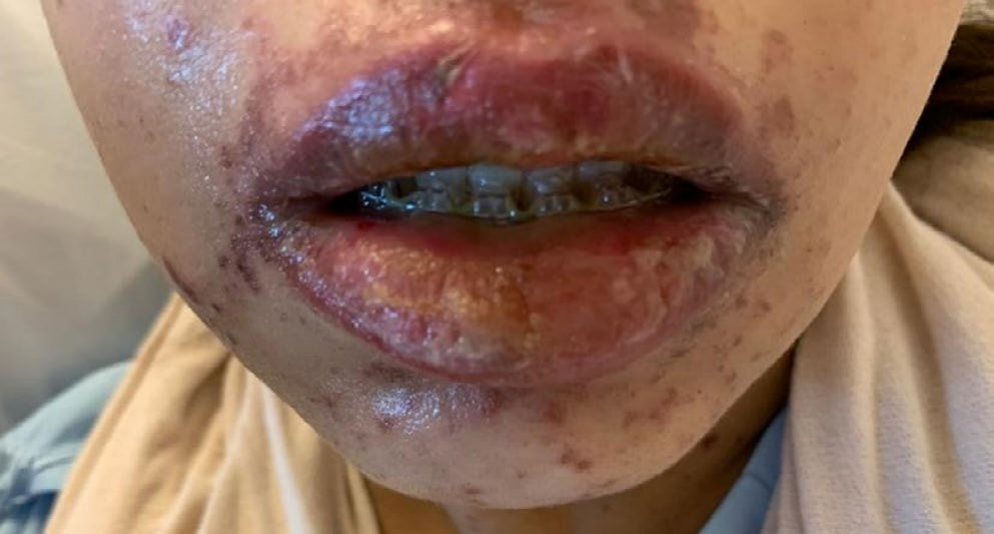
Erythema and erosion on both lips

A 42‐year‐old woman was presented to the hospital with history of methotrexate‐induced pruritus and severe skin reaction. She had ectopic pregnancy 2 years ago and was treated with methotrexate after which she developed severe stomatitis, leucopenia, and severe inflammation of urinary bladder, diagnosed as mast cell activation syndrome at that time. Recently, she was admitted to the critical care unit as a case of SJS with fever, generalized macular rash, buccal ulceration, and burning sensation in her eyes. Further history revealed that she started treatment with carbamazepine 2 weeks before admission treating trigeminal neuralgia. The medical history was otherwise unremarkable. On physical examination, there are several flaccid and ruptured bullae on the Rt hand, back, and legs, and generalized maculopapular rash with target lesions all over the body in centrifugal distribution (Figures 1 and 2). Total area of skin involvement was <10%. Nikolsky's sign was positive (Figure 3). There are erythema and painful erosions on both lips (Figure 4). Patient complain of odynophagia but able to swallow some liquids with involvement of genital mucosa.

Laboratory investigations showed mild leukopenia, no eosinophilia, and thrombocytopenia with mildly elevated aspartate aminotransferase (AST), alanine aminotransferase (ALT), and C‐reactive protein (CRP). No symptoms or signs of infection were found with negative blood, urine, and sputum cultures. No skin biopsy was taken.

Patient was admitted to the critical care unit, and carbamazepine was discontinued immediately; patient received intravenous fluid maintaining positive balance, nutritional support, eye care, and wound care.

Steroid treatment was given for 5 days in the form of 40 milligram methyl prednisolone daily. On the 10th day, patient was discharged.

**Laboratory results**

**Table:** 

Blood test results
WBCs (white blood counts) = 3600
HB (hemoglobin) = 13.4 g
Platelets = 103 000
Urea = 15 mg/dL
Creatinine = 1 mg/dL
ALT = 61.9 U/L
AST = 55.1 U/L
GGT = 109 U/L
CRP = 47.3 mg/L

## 2 DISCUSSION

Stevens‐Johnson syndrome (SJS) is a severe mucocutaneous reaction, most commonly triggered by medications and infection, and in 1 of 3 cases, no cause was identified. There are extensive necrosis and detachment of the epidermis.^1^

Mucous membranes are usually affected in more than 90% of cases. Both SJS and TEN are distinguished chiefly by severity, based upon the percentage of blisters and erosions.^2^, ^3^

Medications are main trigger of SJS, especially allopurinol and antiepileptic medications.^4^, ^5^

The pathology of Stevens‐Johnson syndrome is incompletely understood. Studies suggested a cell‐mediated reaction against keratinocytes leading to necrosis.^6^

Drugs can stimulate the immune system by binding to the major histocompatibility complex (MHC) class I and the T‐cell receptor. The hallmark of SJS is the keratinocyte necrosis, ranging from partial‐ to full‐thickness necrosis of the epidermis.^7^, ^8^

For patients with suspected drug‐induced SJS, withdrawal of the offending agent may improve the prognosis. In one observational study of 113 patients with SJS, early drug withdrawal reduced the risk of death by 30 percent for each day before the development of blisters and erosions.^9^

The main lines of management include fluid and electrolyte management, wound care, nutritional support, pain control, temperature management, and treatment of infections.^10^, ^11^

There are no definitive therapies for SJS.^12^, ^13^ Several immunosuppressive or immunomodulating therapies have been used in clinical practice, including systemic corticosteroids, intravenous immune globulin (IVIG), cyclosporine, plasmapheresis, and antitumor necrosis factor (TNF) monoclonal antibodies.

None of these therapies have been adequately studied in randomized trials except thalidomide, which was found to be harmful.^14^

The use of systemic corticosteroids in patients with SJS has not been evaluated in clinical trials and remains controversial.^15^

Another immunologically medicated disorders is mast cell activation syndrome (MCAS), which is one of mast cell disorders present with signs and symptoms that are caused either by activation of mast cells or by mast cells infiltrating organs.^16^

Mast cell activation syndrome (MCAS) was first proposed as a distinct idiopathic disorder in 2010.^17^ Subsequently, the definition of MCAS expanded to also include primary and secondary categories, making "mast cell activation syndrome" essentially an umbrella term that describes a clinical presentation, rather than a specific diagnosis.^18^

In our case, the patient was diagnosed earlier with mast cell activation syndrome with pruritus and severe skin reaction; 2 years later, the patient was prescribed carbamazepine treating trigeminal neuralgia; the history of drug‐induced immunologically mediated mast cell activation with skin pruritus was missed; and patient developed severe form of SJS Good history taking is crucial if treatment with carbamazepine is clinically indicated. We would like to alert all physicians that carbamazepine should be avoided in any patient with a previous history of drug reaction such as mast cell activation syndrome.

## 3 CONCLUSION

Stevens‐Johnson syndrome (SJS) is very serious skin condition. Carbamazepine is one of the common medications that can cause (SJS). Good history taking is crucial if treatment with carbamazepine is clinically indicated. Physicians should be alert to avoid carbamazepine in any patient with a previous history of drug reaction such as mast cell activation syndrome.

## CONFLICT OF INTEREST

The author(s) declared no potential conflicts of interest with respect to the research, authorship, and/or publication of this article.

## AUTHOR CONTRIBUTIONS

TZ: wrote the article. ZIB and OSM: shared in the discussion. MA: collected data and revised the manuscript. All authors reviewed the final draft of the manuscript and approved its submission.

## CONSENT

Informed consent was obtained from the patient for the publication of this clinical image.
